# Fully endoscopic stapes surgery (stapedotomy): technique and preliminary results

**DOI:** 10.1590/S1808-86942011000600008

**Published:** 2015-10-19

**Authors:** João Flávio Nogueira, Marcos Jullian Barreto Martins, Carolina Veras Aguiar, Antônio Israel Pinheiro

**Affiliations:** 1 ENT Physician; 2 3rd Year ENT Resident; 3 3rd Year ENT Resident; 4 ENT Physician

**Keywords:** ear ossicles, endoscopes, otosclerosis, stapes surgery

## Abstract

**Abstract:**

Stapedotomies are perfomed with the aid of surgical microscopes. However, the microscope has some limitations and may cause complications such as damage to the chorda tympani nerve. There are just a few cases and no series published on the use of sino-nasal endoscopes in stapedotomies.

**Objectives:**

a) To investigate the feasibility of using sino-nasal endoscopes in stapedotomies, focusing on the visualization of important structures. b) To show initial results, discuss possible advantages and disadvantages of this instrument.

**Methods:**

15 patients with otosclerosis were selected to undergo stapedotomies in 2010. The data and surgery videos were analyzed retrospectively (study case series). The anatomical conditions of the oval window niche and surgical steps were described and used to assess possible benefits of such procedures.

**Results:**

The surgeries were performed with endoscopes only and all relevant anatomical structures were visualized without difficulty. No complications were observed and 14 of the 15 patients reported improvement of their hearing, confirmed by postoperative audiological tests.

**Conclusion:**

Totally endoscopic stapes surgeries are technically feasible, safe and promising. In this small series, the main advantages were: virtually no trauma to the chorda tympani nerve and excellent vision. The disadvantages were the lack of stereoscopic vision, having to work with one hand only and the learning curve.

## INTRODUCTION

Stapedectomies and stapedotomies are currently done in most centers in the world under the microscope. Although many have been the techniques already described in recent years, results continue to be excellent with very low risk of complications^1^, ^2^, ^3^.

Surgical microscopes provide a good quality amplified image in a straight line. However, such limitation inherent to the equipment limits visual field when we make exclusively transcanal access in the narrowest segment of the external ear canal. In winding canals, this may represent an even greater limitation, requiring other access pathways to the middle ear, behind the ear or modified transcanal approaches^3^,^4^.

Even with extended access to the middle ear, one of the important steps in performing stapes surgeries under the microscope is to partially remove the bone wall of the most medial segment of the external acoustic meatus. This important step in this surgery enables a better exposure of the incus-stapes joint, the oval window niche, pyramidal eminence and other important structures in this procedure.

Nonetheless, such step requires exposure, manipulation and, in some cases, irreversible trauma of the chorda tympani nerve in order to have the best visualization of the oval window niche.

Another important point to be considered during stapes surgery under the microscope is the visualization of the stapes supra-structure. Most of the times, when using a transcanal approach under the microscope, the surgeon is unable to see the stapes anterior crus, forcing the surgeon do blindly fracture such structure.

Although endoscopes were introduced in ear surgeries over 15 years ago, their role have been rather limited in the treatment of middle ear inflammatory disorders and otosclerosis^1^, ^2^, ^3^, ^4^, ^5^.

There are many reasons for this, such as the belief of a limited and marginal role endoscopes play in middle ear surgery, instrument limitation, and a potentially long learning curve in order to get used to single-handed work and the lack of a stereoscopic view^2^,^3^.

There already are some reports concerning the use of endoscopes in ear surgeries, including stapes surgery; however, the all discuss the use of otological endoscopes, 3mm in diameter and 10cm in length. Due to its limited diameter, these endoscopes have a very restricted visual field, differently from the regular endoscopes used in sinonasal surgeries^2^, ^3^, ^4^.

Sinonasal scopes with 4mm in diameter and 18cm length, with wide-angle lens and different angles, allow for an amplified image which can be quickly modified by advancing or pulling the instrument back^2^. And, differently from the shorter otologic endoscopes, these longer instruments provide a larger field for working with one's hands, since the hand holding the endoscope does not interfere with the other hand holding the surgical instruments.

The goals of the present study are:

a) To assess the possibility of using 4mm and 18cm endoscopes in fully endoscopic stapes surgeries.
b) To show the preliminary results, assessing the possible pros and cons of using these instruments.

## MATERIALS AND METHODS

After approval by the Institution's Ethics Committee (IOF 01/2010), 15 patients were selected to undergo stapes surgeries between January and July of 2010.

Inclusion criteria were: patients diagnosed with otosclerosis - based on clinical history, straight external ear canal, normal otoscopy and with audiometric tests showing bilateral conductive hearing loss with an air-bone gap larger than or equal to 30 decibels (dB), no stapedius reflex, with normal bone conduction values at 500, 1000, 1500 and 2000 Hertz (Hz), without a past of middle ear infectious diseases and with normal temporal bone CT scan.

Exclusion criteria were: a past of middle ear infectious disease, very winding external acoustic meatus, changes seen upon otoscopy: such as tympanic membrane perforation and vocal and tonal audiometry showing conductive hearing loss with an air-bone gap less than 30 dB.

All surgical procedures were digitally recorded. Some anatomical situations were assessed in the postoperative from the surgery videos, as follows:

a) Oval window niche exposure;
b) Need to manipulate the chorda tympani nerve;
c) Visibility of the stapes crura (especially the anterior crus);

We assessed the postop audiological results and complications. The audiometric tests were held between 30 and 40 days after the surgeries and the complications were listed according to reports from the patients in medical charts and in the postop visits, held 7, 15 and 45 days after surgery.

### Surgical technique

**Figure 1:**
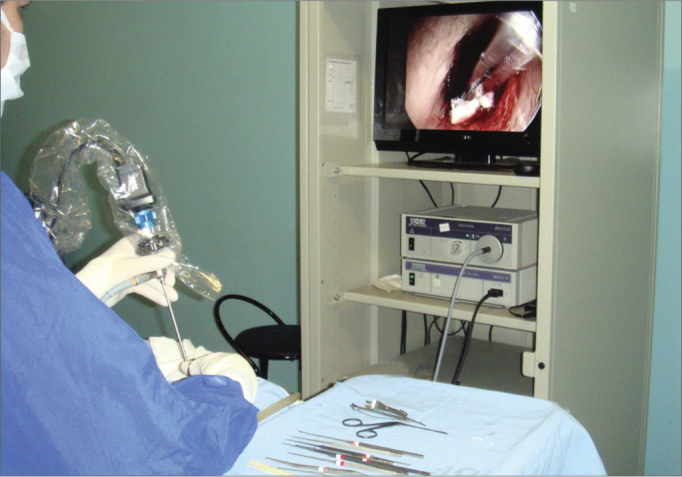
Position in the operating room. The ENT surgeon performs the procedure looking directly at the monitor in front. The instruments must be positioned at easy access, such as on this table next to the surgeon.

All procedures were carried out under hypotensive general anesthesia. The patients were all placed in the same position for conventional ear surgeries done under the microscope. The video equipment was placed in front of the surgeon (Figure 1). We used basically the same techniques and instruments used in conventional ear surgeries, except for the use of the 4mm in diameter and 18cm long endoscope with 0 to 30 degrees, curved micro aspirators and curved-tip micro scissors.

Cotton balls soaked in 1:2000 adrenalin were placed in the external acoustic meatus and left there for 5 minutes. We did not inject locally.

**Figure 2:**
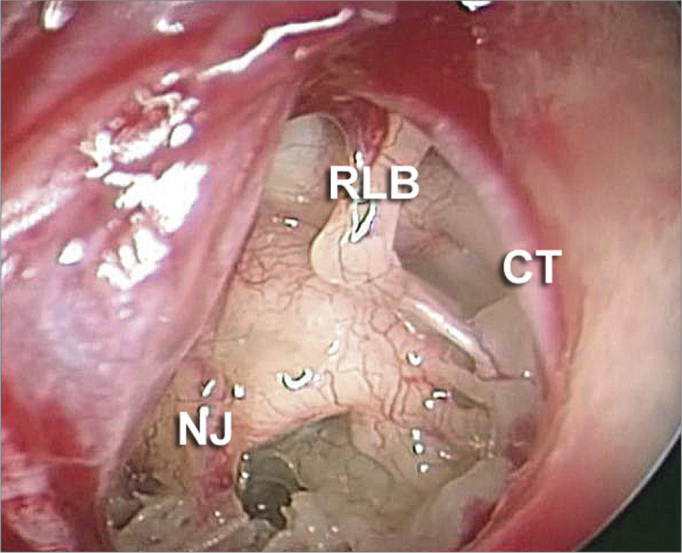
Endoscopic image from a 0 degree scope after making the tympanic-meatal flap. We can see important middle ear structures, such as the long process of the incus (RLB), chorda tympani nerve (CT) and in some cases, we can also see the Jacobson nerve (NJ).

With the 0 or 30 degree angle endoscope we raised a tympanic-meatal flat in the posterior region of the external acoustic meatus. We carried out vertical (6 and 12 hours) incisions and one horizontal incision which merged them posteriorly at about 1 to 1.5 cm from the tympanic membrane. After raising the flap we inspected the middle ear with the 30° endoscope (Figure 2). We paid special attention to the position of the facial nerve, probing for a possible positioning or a prolapse of the nerve over the stapes footplate.

A dedicated test with the ossicular chain was done in order to check for stapes fixation. The stapes tendon was cut with a micro-knife or a curved micro-scissors and the incus-stapes joint was cut.

**Figure 3:**
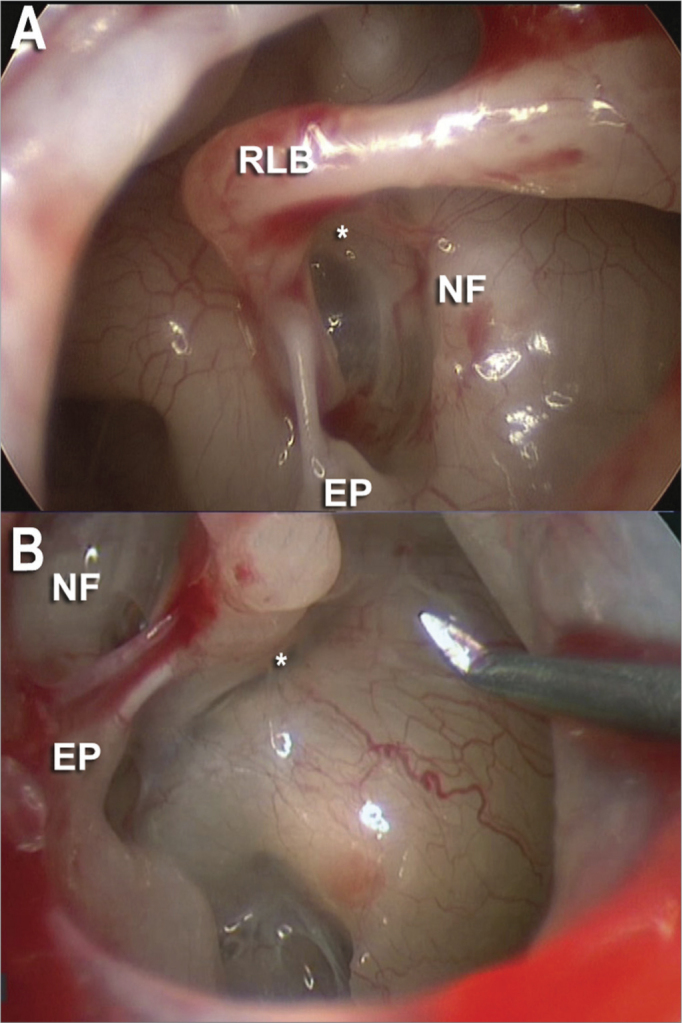
30 degree scope endoscopic view of the oval window niche. A: facial nerve canal (NF), long process of the incus (RLB), pyramidal eminence (EP) with its tendon and its anterior and posterior crura (*). B: In another case we noticed the same endoscopic view with the 30 degree scope.

The stapes superstructure was carefully fractured in the anterior and posterior crura under direct endoscopic view (Figure 3) and removed, leaving the footplate fully exposed. In all the cases we used a Teflon prosthesis (0.6mm in diameter and 6mm in length), which was made from the length measured between the footplate and the medial surface of the incus's long process.

After this measurement, which is usually 4.5mm, we cut the prosthesis using the surgical ruler (scaled in millimeters) and the #11 scalpel blade. At this step, the endoscope was positioned outside of the surgical field.

**Figure 4:**
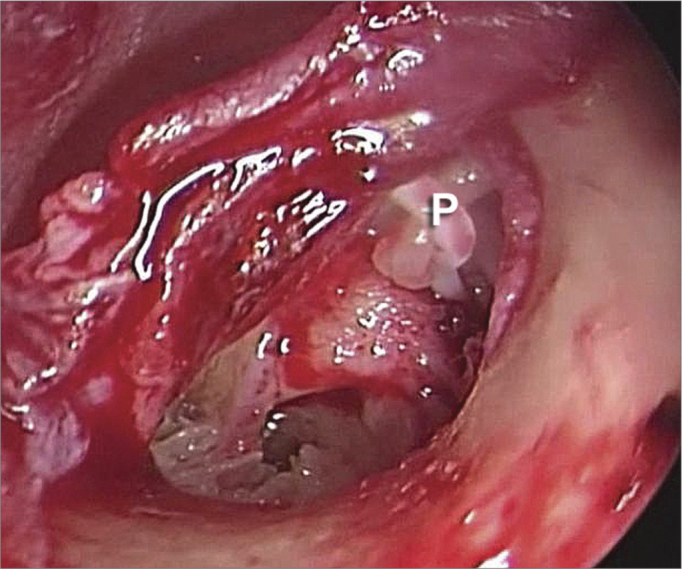
Endoscopic view (30 degree instrument) of the Teflon prosthesis (P) in place.

After cutting, the endoscope was repositioned in the surgical field, and a small, 0.6mm in diameter, hole was punched in the posterior portion of the stapes footplate, with a small 0.6mm tip perforating instrument. The prosthesis was placed in this hole and fit along the long process of the incus (Figure 4).

In order to properly position the prosthesis, we previously opened its fitting point in the long process of the incus with a small stylet or a micro forceps. After such opening, with the micro-forceps, we tried to place the prosthesis directly in the hole created in the stapes footplate. After positioning it in the hole, with the stylet we fit the prosthesis to the long process of the incus. Since we used a Teflon^®^ prosthesis, a material which does not have memory, it was not necessary to tighten the prosthesis in the long process of the incus.

We avoided frequent suctions, especially after opening the footplate, in order to avoid post-op complications, such as vertigo and cochlear damage. The malleus was palpated in order to rule out head fixation and make sure the entire ossicular chain moves all the way to the prosthesis. In order to seal the footplate, we used small pieces of dry Gelfoam^®^, placed with the micro scissors. Following that, the tympanic-meatal flap was repositioned and we then inserted Gelfoam^®^ dressing in the external acoustic meatus, without ointments or creams.

## RESULTS

We did the fully endoscopic stapes surgery in all 15 patients, two men and 13 women, with average age of 36.86 years. In all the patients, the procedure was carried out through the external acoustic meatus, using the 4mm and 18cm long endoscope, of 0 and 30° angle.

Analyzing the surgical endoscopic view of the oval window niche, we noticed:

a) With a 0 degree endoscope, in eight of the 15 patients (53.3%), we obtained an ideal exposure of the oval window niche, of the facial nerve segment and also of the pyramidal eminence.
b) With a 30 degree scope, in 12 of the 15 patients (80%), we achieved an optimal exposure of the oval window niche, the facial nerve segment and the pyramidal eminence.

As to the manipulation of the chorda tympani nerve, there was a need to manipulate it in three cases (20%). In these patients, when we raised the tympanic meatal flap, the chorda tympani nerve was in front of the visual field, preventing a proper view of the oval window niche.

As to the exposure of the stapes anterior crus (Figure 3) its fracture was achieved in all cases through direct endoscopic view, using the 30 degree scope.

We did not have any intraoperative complication, nonetheless, there was some difficulty in properly positioning the prosthesis.

One patient, in whom we had to manipulate the chorda tympani nerve, had a temporary reduction in his taste sense. No specific medication was prescribed for this patient, who reported substantial improvement in his postop visit, 15 days after the procedure.

Another patient, in whom we had difficulty in placing the prosthesis, there was postop vertigo. The patient was medicated and was also symptom-free in his postop visit 15 days after the procedure.

There were no postoperative tympanic perforations, nor external acoustic meatus hematomas.

**Figure 5:**
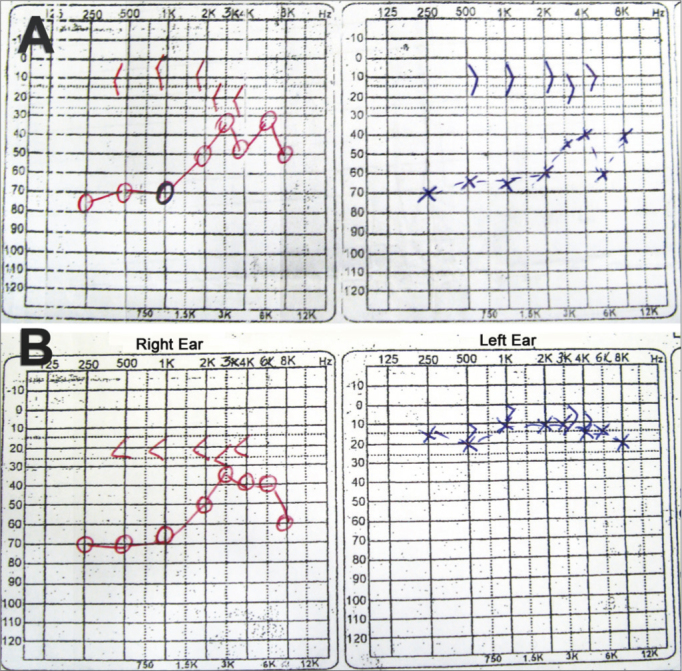
Fax of the audiogram belonging to the patient submitted to left ear fully endoscopic stapes surgery. A: preoperative test showing the conductive-type curve bilaterally. B: post-op test showing the conductive hearing loss in the right ear and within normal ranges in the left ear.

As far as the audiologic results are concerned, we noticed a subjective hearing improvement in all patients upon their return visit 15 days after surgery. 14 patients (93.3%) had audiogram improvements. In these, the air-bone levels were similar, within normal standards in the previously studied frequencies (500; 1,000; 1,500 and 2,000 Hz) (Figure 5).

In one patient (6.7%), there were no similarities between air and bone levels in the frequencies considered, and there was still a maximum air-bone gap of 25 dB in these frequencies.

The mean preoperative SRT value the patients had was 65 dB and the postoperative SRT was 25 dB.

## DISCUSSION

Since the introduction of the classical stapes surgery by Shea, many variations have been described in the literature. However, they all use the surgical microscope^6^.

**Figure 6:**
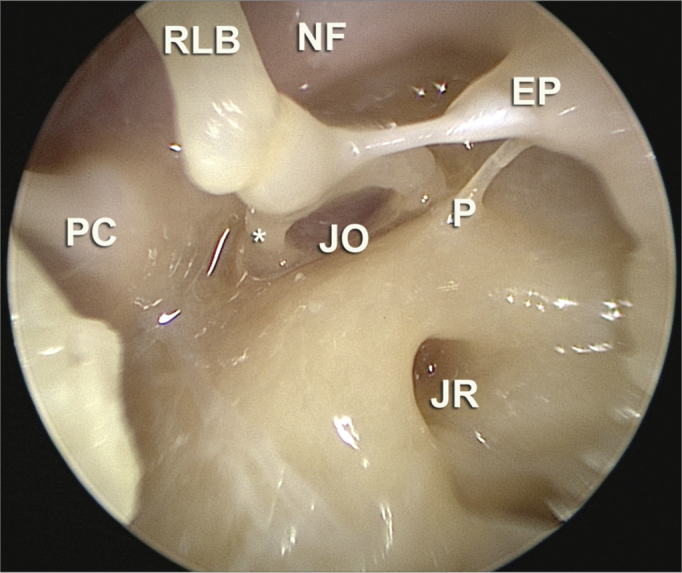
Endoscopic image of the anatomical dissection after removal of the ear drum, with a 30 degree scope, showing the type of image which can be achieved by means of endoscopic approaches. Here we see the niches of the round and oval windows (JR and JO, respectively), pyramidal eminence (EP), ponticulus (P), facial nerve canal (NF), long process of the incus (RLB) and incus-stapes joint; also the cochleariform process (PC) and practically the entire posterior tympanic sinus.

Endoscopes are being increasingly used in middle ear surgery. Many anatomy-based papers have been published, showing the possible advantages of using endoscopes in ear surgery, especially due to a better visualization of the structures and recesses within the tympanic cavity (Figure 6).

These papers bring about a new approach for known disorders, such as the cholesteatoma, introducing new physiological and anatomical concepts concerning aeration of the tympanic cavity and mastoid cells in order to understand the disease and its surgical treatment^7^, ^8^, ^9^.

Today, the endoscope plays an important role in cholesteatoma surgery in some centers in the world; however, there are still very few papers in the literature discussing the possible benefits of stapes surgeries made solely with the use of the endoscope, especially those studies based on the exposure of the oval window niche and stapes visibility.

Based on endoscopic anatomic studies in the middle ear of cadavers^9^, ^10^, ^11^, ^12^, we started to do stapes surgery with the endoscope only, a transcanal approach with a 4mm in diameter and 18cm long endoscope.

Although we did not do any comparative study, this endoscopic approach seems to provide some advantages, such as a better view during the procedure and also a better view of important structures in the middle ear, especially that of the oval window niche, without a need to remove bone.

When we perform stapes surgery under the microscope, in almost all the cases there is the need for a partial removal of the medial bone segment on the posterior wall of the external acoustic meatus. This removal can be made by using delicate curettes or a burr with a micro motor. However, this implies some degree of chorda tympani nerve manipulation, or even totally damaging it. In some patients, this maneuver may be more difficult for anatomical reasons, causing damage to the facial nerve when further burring is needed.

In the fully endoscopic stapes surgeries from our series, we obtained excellent visualization with the 30 degrees angled endoscope in the oval window niche, the pyramidal eminence, facial nerve and long process of the incus, without the need to cut or burr this posterior wall of the external acoustic meatus. Nonetheless, three patients had their chorda tympani nerve manipulated, because when we raised the tympanic-meatus flap, this nerve was right in front of our visual field, preventing us from seeing the oval window. We handled the nerve very carefully, but even then one patient complained of temporary taste changes.

That fact that it is not in all cases that we have to burr or curette the posterior wall of the external acoustic meatus, and very little or no touching the chorda tympani nerve, seems to be one of the pros of the endoscopic technique when compared to the microscopic approach. It may be because of the fact that we do not curette and also because we make broad tympanic-meatal flaps, there were no patients with postop tympanic perforations.

Another interesting point, it is a single-handed work, which is necessary during fully endoscopic stapes surgery, since the surgeon holds the endoscope with one hand and works with the other. During stapes surgeries done under the microscope, the surgeon holds the instruments with one of the hands, and the ear speculum with the other. And this does not preclude excellent results.

In our limited series, although we did not take notes about the surgery, in the first cases we had adaptation difficulties and also with some movements, especially when placing the prosthesis in its proper place.

In this preliminary study, limited to 15 cases only, without any revision patient, we noticed that the endoscope provided a better view of the stapes anterior crus, enhancing the fracturing maneuver under direct view and no longer done blindly, as it is done in most of the microscope approach cases.

In this limited series, we have used, so far, the same traditional ear surgery instruments. Except for the curved micro scissors and curved micro aspirators, the other instruments were the same. However, it is worth stressing that a new set of instruments must be developed for fully endoscopic stapes surgery, as well as for other endoscopic ear surgeries.

We used the same endoscopes used in nasal surgeries, with 4mm in diameter and 18cm long. These instruments may provide some advantages when compared to ear endoscopes, such as better field of vision and better image quality, since there is better light transmission due to the very gauge of the instrument.

**Figure 7:**
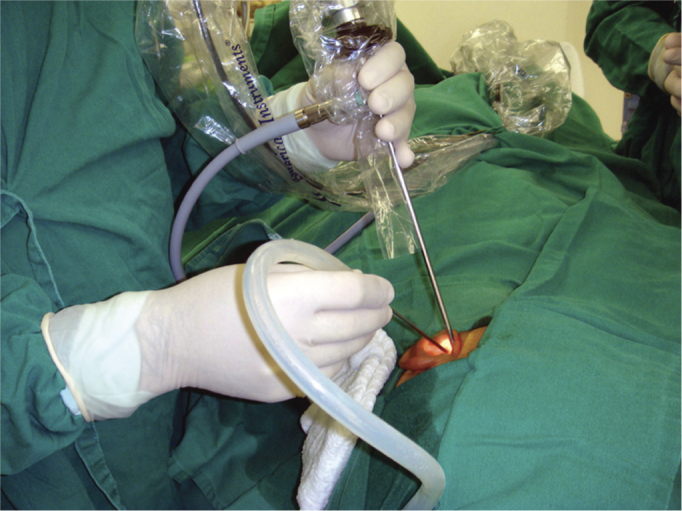
Figure showing how the endoscope is positioned inside the external auditory canal, as well as hand position - one holding the endoscope and the other holding the instruments. Notice that the hands are in different horizontal planes.

We chose these endoscopes for two reasons:

a) Availability: these endoscopes are available practically in all hospitals which routinely perform ENT surgeries;
b) Ease of operation: since they are long, these endoscopes do no interfere in the task, nor with hand movement, they remain in different horizontal planes (Figure 7), differently from what happens with traditional ear endoscopes.

Nonetheless, there are some safety aspects which must be observed:

a) Heat: The tips of nasal endoscopes tend to directly transfer the heat generated by the light source. Special attention must be paid to the tip of the endoscope, since it may have a high temperature, which may damage some structures.
b) Accidental endoscope movements: this may cause accidental trauma by the tip of the endoscope upon important structures in the middle ear and in the external acoustic meatus. In the meatus, special care must be taken in order to avoid unnecessary bleeding caused by trauma in the region. In the middle ear, special care must be taken considering all potentially important structures, such as the ossicular chain, the facial nerve, and others. To help avoid such trauma, the surgeon must keep the endoscope in the surgical field when not looking directly to the screen.

As far as audiological results are concerned, there was auditory improvement, with closure of the air-bone gap in the frequencies considered (500; 1,000; 1,500 and 2,000 Hz) in 14 of the 15 patients submitted to surgery. In one patient, despite his subjective improvement in hearing, it was not confirmed by the audiometric exam carried out between 30 and 45 days after the surgeries. So far, we do not know the real cause of such hearing loss, but we believe the prosthesis may have shifted.

In a clear contrast to the impact of introducing the endoscope in most medical specialties, the ear surgery practice has changed very little in recent years, the microscope still prevails.

However, depending on the need, it is likely that there are some instances in which the endoscope could be the best instrument when compared to the microscope, and vice-versa^3^.

Otorhinolaryngologist must try to work and learn to handle both types of instruments, endoscopes and microscopes, in order to better understand and treat ear diseases, increasingly benefitting patients.

## CONCLUSION

In the present limited series, we show that it is possible to perform stapes surgery using only the 4mm in diameter and 18cm long endoscopes of different angulations, without major difficulties.

Audiological results show that there was proven hearing improvement in the 14 of the 15 patients. We had no complications.

The main advantages of a fully endoscopic stapes surgery were: no handling and, consequently, no damage to the chorda tympani nerve in almost all the patients, and excellent visualization of the oval window niche, stapes anterior crus and its suprastructure. The main disadvantages were: need to work with one hand only, no stereoscopic vision and a learning curve.

Nonetheless, more cases and a longer follow up period are mandatory to assure an important role for endoscopes in stapes surgeries, as well as in other ear surgeries.
